# Open questions: The disrupted circuitry of the cancer cell

**DOI:** 10.1186/s12915-014-0088-y

**Published:** 2014-10-18

**Authors:** H Steven Wiley

**Affiliations:** Pacific Northwest National Laboratory, 902 Battelle Boulevard, K8-96, Richland, WA 99352 USA

Every new decade of biology brings with it a change in outlook driven by new technologies and fresh perspectives. Such is the case for cancer and how we consider the disease. The advent of molecular biology led to the identification of altered signaling molecules and 'oncogenes' that were proposed to drive uncontrolled cell proliferation [1]. The rise of cell biology and new imaging and culturing technologies led to the idea that disruptions in the extracellular environment prime cells for transformation [2]. In the current genomics era, cancer is most commonly seen as a genetic disorder where an unstable genome gives rise to a variety of different cell variants that are selected for proliferation and survival [3]. All of these views are partially correct, of course, and are simply different ways of saying that genetic alterations in cancer cells result in a loss of growth homeostasis. They also take the view that molecular changes 'drive' a cell to grow uncontrollably, rather than tip the balance from one normal state (quiescence) to another (proliferation). Underlying this oversimplification is a profound ignorance of what controls homeostatic cell growth in the first place and how specific mutations impact it.

Normal, proliferation-competent cells can accurately monitor their environment and respond appropriately to perturbation, whether it is a loss of neighbors or an inflammatory stimulus. Cancer cells either proliferate or refuse to die where and when they should not, which clearly indicates that they have problems in detecting or responding to their environment. Thus, an enormous amount of effort has gone into defining the signaling pathways that can trigger a proliferative response and the biochemical mechanisms underlying these pathways. Far less work has focused on understanding the higher-order logic of these pathways and the roles played by all of the components as part of an integrated system. In other words, we do not really understand how cells process information and make decisions and thus cannot predict how any given molecular change will alter what a cell does.

## Cells as an information-processing machine

Consider the cell as a computer that performs some useful task that we wish to modify. We know that typing on a computer’s keyboard will result in the execution of specific tasks, so we focus on how keyboards work. Intensive studies will reveal how keystrokes are turned into electrical signals and routed to different parts of the computer, but will never tell us how the CPU interprets the keystrokes as commands or executes some specific program or subroutine. This same type of knowledge is crucial for a full understanding of how cancer operates. A cancer cell receives essentially the same contextual information as its normal counterparts, but it processes it in an aberrant way so that it executes the wrong program (for example, proliferation). Our current knowledge is allowing us to diagnose faulty 'keyboards' that send wrong commands, but not faulty programs or defects in the cellular 'CPU'. Such errors in information processing undoubtedly underlay a large fraction of cancers.

Most of the work that has been done on cell information processing has focused on transcriptional regulation, mostly because of easily quantified endpoints (levels of a specific transcript), as well as the availability of simple model systems, such as yeast cells, that can be readily manipulated. However, the connection between levels of transcripts and the functional state of the cell is complex and poorly defined. In addition, connections between the different signal input streams and transcriptional activation are poorly understood. Recent work that integrates different types of’omics data to understand gene regulatory patterns showed that cells respond to changes in their environment through a complex network of genes and proteins, but the 'logic' of these networks is not apparent [4]. It remains an open question whether cell circuits will ever be definable in terms of some logical architecture or whether their distributed nature will always require computational tools to understand them.

We do know that information processing by cells is distributed between multiple intercellular components, such as the cytoskeleton and cell surface receptors, and can depend on multiple molecular processes that occur at different scales. For example, specific cell responses are highly dependent on an extracellular network of interactions between specific environment components, such as the extracellular matrix and the presence of specific cell types. This extracellular information-processing network is also highly dynamic. Cell-secreted proteases can activate growth factor precursors or inactive complexes. Conversely, secreted binding proteins can block the activity of previously active extracellular factors [5]. Extracellular information processing can have a profound effect on cancer progression. For example, CSF-1 produced by breast cancer cells can cause local macrophages to release epidermal growth factor that is necessary for their proliferation and migration [6]. What is unknown, however, is the role of such dependencies in normal cell functions.

## What is normal?

One of the hallmarks of cancer is a loss of the cancer cells’ dependency on specific growth factors for growth [1]. When it was discovered that some cancer cells make their own growth factors in a process known as autocrine signaling, it was thought that this could be a primary mechanism underlying cancer. It turned out, however, that most normal cells also undergo autocrine signaling as part of their context-detection mechanism [7]. Still, interrupting these 'normal' processes has been shown to be an effective way to inhibit the growth of cancer cells, suggesting that although aberrant in their overall growth phenotype, cancer cells are still highly dependent on many of the same signaling pathways as their normal counterparts. Indeed, the degree to which cancers arise from a quantitative imbalance in normal feedback processes rather than a defect in a central signaling pathway is a critical issue to address.

One of the essential architectural features of all information processing systems is feedback. Signaling pathways are known to display numerous positive and negative feedback loops that combine to define their overall functional output. Depending on their strength and pattern of interconnections, these feedback loops can give rise to a wide range of signaling processes, such as switch-like or graded responses or even oscillations [8]. The plethora of feedback mechanisms associated with even the simplest signaling system suggests that these are crucial for their information processing functions. Indeed, autocrine signaling has been shown to be part of a positive feedback system that integrates multiple extracellular signals into a single output [9]. Feedback also provides robustness and stability to signaling systems, by preventing too much signaling by overactive receptors and moderating the effect of potential inhibitory drugs [10]. From a conceptual level, dysregulation of feedback control systems would seem necessary for the development of cancer, yet this area of research has scarcely been explored.

One of the problems with investigating feedback control is that it is exceedingly difficult to design, execute and interpret experiments that modify it. Because feedback control is intrinsically dynamic, the responsible proteins, such as phosphatases or proteases, tend to be expressed at low levels and rapidly turn over [11]; thus, they are hard to detect. Feedback can also give rise to non-linear effects dependent on its pattern and magnitude, making it almost impossible to predict the effect of a perturbation unless the system is extremely well characterized and controlled. It is thus far easier to study the core components of signaling pathways, even if they are not the most relevant players. It is always easier to look for lost keys under the streetlight.

Despite the difficulties in studying recursive feedback systems and the consequent paucity of data on the role they play in cell information processing, genetic studies have shown a strong correlation between loss of key feedback regulators and cancer [12]. It will be critical to understand the roles they play in both normal and cancer cells, not only because of the impact of their loss, but because manipulating them provides potential new avenues for more effective cancer therapeutics.
